# User Acceptance of an AI-Powered Medical History–Taking Training System Among Undergraduate Medical Students: Mixed Methods Study

**DOI:** 10.2196/101425

**Published:** 2026-07-21

**Authors:** Yang Liu, Yiying Zhu, Chujun Shi, Xian Lu, Liping Wu, Minghui Yue, Xiaolin Hong, Oudong Xia, Weishan Zhang

**Affiliations:** 1 Medical Simulation Center Shantou University Medical College Shantou China; 2 Department of Medical Physics and Informatics Shantou University Medical College Shantou China; 3 Shantou University Medical College Shantou China

**Keywords:** artificial intelligence, Kirkpatrick framework, medical education, medical history-taking, mixed methods, user acceptance, virtual patient

## Abstract

**Background:**

AI-powered virtual patient systems provide medical students with repeatable practice environments for history-taking training. However, user acceptance of such systems and the experience dimensions associated with that acceptance lack mixed methods evidence.

**Objective:**

This study aimed to (1) examine the associations of system experience and learning experience/intrinsic motivation with overall acceptance among undergraduate medical students using an AI-powered medical history-taking training and evaluation system (AMTES), (2) explore user experience patterns through open-ended questions, and (3) integrate quantitative and qualitative findings to inform system refinement and pedagogical implementation.

**Methods:**

A cross-sectional convergent mixed methods design was used. A total of 66 undergraduate medical students at a Chinese medical college completed a postuse questionnaire after AMTES training. The primary outcome was an overall acceptance composite combining use intention, recommendation intention, and overall satisfaction. Associations with system experience and learning experience/intrinsic motivation were examined using linear regression with heteroscedasticity-consistent SEs (type 3) and bootstrap CIs. Sensitivity analyses included covariate adjustment, single-outcome models, a fractional logit model, and content-overlap sensitivity checks for the system-experience composite. Open-ended responses were analyzed using codebook-oriented thematic analysis and integrated with quantitative findings through a joint display.

**Results:**

Both system experience and learning experience/intrinsic motivation were positively associated with overall acceptance (standardized β=.526; *P*<.001 and standardized β=.377; *P*=.002, respectively; *R*^2^=0.662). Sensitivity analyses supported the robustness of both positive associations. Qualitative analysis showed that the most frequently nominated benefits clustered within the “Practice Accessibility and Feedback Support” theme, particularly self-directed practice (28/66, 42.4%) and immediate feedback (25/66, 37.9%). Refinement priorities clustered around 4 dialogue-quality and assessment dimensions, including semantic understanding, contextual consistency, conversational naturalness, and scoring logic, each cited by 21%-35% of respondents. The mixed methods joint display indicated contextual (same-source) alignment on dialogue and scoring concerns and suggested that perceived scoring-feedback discrepancies may be associated with lower student trust in the feedback function. This cross-dimensional pattern was not apparent from the quantitative model alone.

**Conclusions:**

In this exploratory cohort of undergraduate medical students, both system interaction quality and perceived learning value were positively associated with overall acceptance of AMTES, with system interaction quality showing the stronger association within the study’s measurement specification. Dialogue coherence, semantic understanding, and scoring-feedback alignment emerged as the most frequently nominated refinement priorities and are plausible candidate targets for improving acceptance-related perceptions. This study emphasizes implementation-level acceptance rather than solely technical reliability or educational effectiveness. Interaction-quality problems may be associated with less favorable acceptance even when learning value is recognized. These findings may inform system refinement priorities and the curricular integration of AI-powered history-taking training systems, while larger multicenter and longitudinal studies are needed to examine whether improvements in interaction quality translate into gains in learner acceptance and downstream training outcomes.

## Introduction

History-taking is a fundamental clinical skill, and its quality directly affects diagnostic accuracy [1,2] and subsequent clinical reasoning [1]. For medical students, mastering a complete history-taking process and developing fluent clinical interview skills are core tasks in the transition from classroom learning to clinical practice [3]. However, achieving this transition has become increasingly difficult, as the erosion of bedside teaching and the decline of core clinical skills have been recognized as growing challenges in medical education [4]. Simulation-based instruction and standardized patient (SP) encounters have been proposed as effective alternatives to address this gap, with evidence supporting their value in improving student confidence and clinical competence [5,6]; however, their systematic implementation remains constrained by time and resource demands, limiting opportunities for repeated practice and structured feedback [5].

Virtual patient (VP) systems have emerged as a promising approach to address these challenges, offering interactive and repeatable practice environments suited to clinical reasoning training [7]. Existing evidence supports their educational value: a systematic review of conversational VP interventions found consistent improvements in history-taking and clinical reasoning competencies across multiple studies [8], and a single-cohort intervention study demonstrated statistically significant gains in both self-reported competence and confidence, corroborated by improved objective structured clinical examination performance in medical and surgical history-taking [9]. Notably, that same systematic review found that student satisfaction tended to be higher when VP systems incorporated AI and natural language processing to enable more realistic conversational interaction [8], suggesting that AI-enhanced dialogue may be associated with more favorable learner experiences compared with scripted or menu-driven formats. Building on this trajectory, recent advances in large language models have enabled a new generation of AI-powered virtual patient (AI-VP) systems capable of open-ended natural language interaction and automated feedback [10-12]. However, empirical evidence specifically evaluating AI-VP systems in history-taking education remains limited and preliminary, and a recent scoping review of AI applications in medical education underscored both the promise of these technologies and the need for rigorous evaluation [13]. Sustainable adoption therefore depends not only on technical performance and educational effectiveness, but also on user acceptance [14,15].

From a training-evaluation perspective, Kirkpatrick’s 4-level model provides a useful framework for considering how learners respond to educational interventions. Its first level, “Reaction,” focuses on learners’ immediate responses to training, including satisfaction and perceived value, and is sometimes considered an early indicator of subsequent learning engagement, though the assumption that Level 1 reactions predict higher-level outcomes has been critically examined in medical education [16,17]. Nevertheless, Level 1 evaluation remains a recognized starting point for understanding learner responses to novel educational technologies, particularly in early-stage implementations where downstream outcome data are not yet available. The Technology Acceptance Model (TAM) and related frameworks such as the Unified Theory of Acceptance and Use of Technology (UTAUT) emphasize that perceived usefulness, ease of use or effort expectancy, and related performance beliefs are central determinants of technology acceptance [18,19]. A recent systematic review of TAM in medical education suggested considering broader acceptance frameworks, including TAM2 extensions, for educational technologies in context [14]. Although mixed methods work on trust and acceptance of AI are emerging in medicine [15], research that combines quantitative associations with qualitative exploration of underlying experiential patterns in medical education remains limited. In this study, Kirkpatrick Level 1 provides the curricular-evaluation positioning, whereas TAM/UTAUT-style acceptance logic informs the operational acceptance outcome (use intention, recommendation intention, and overall satisfaction). We treated acceptance as an exploratory, theory-informed construct rather than as a formal test or validated adaptation of TAM or UTAUT, and the experience composites correspond only partially to established acceptance constructs, as detailed in the Methods.

Our team has previously conducted a series of studies on the self-developed AI-powered medical history-taking training and evaluation system (AMTES): study 1 demonstrated the consistency between AMTES automated scoring and human scoring for medical history-taking [20], and study 2 provided preliminary evidence for its educational effectiveness in a real-world teaching environment [21]. Although study 1 reported favorable overall user approval as a secondary finding, neither prior study was designed to explain the determinants of acceptance or identify user-experienced priorities for refinement. Prior work on AI-powered history-taking training systems appears limited, particularly regarding studies that simultaneously examine which experience dimensions are associated with user acceptance and explore the experiential patterns underlying those relationships.

As the third study in this series, this study extends the evidence base to user acceptance, positioned as a Level 1–aligned evaluation of learner reactions after structured curricular use. A convergent mixed methods design was adopted: the quantitative strand estimated associations between 2 theory-informed, empirically constructed experience composites—system experience (interaction quality and ease of use) and learning experience/intrinsic motivation (perceived learning value)—and overall acceptance, while open-ended responses contextualized those associations and identified concrete refinement targets. This study aimed to (1) examine how these 2 dimensions were associated with overall acceptance, (2) explore user-reported experiential patterns underlying perceived benefits and refinement priorities, and (3) integrate quantitative and qualitative findings to generate evidence-informed candidate recommendations for system refinement and pedagogical implementation.

## Methods

### Study Design

This cross-sectional study used a convergent mixed methods design, with quantitative and qualitative data collected through the same postuse questionnaire. Reporting followed the STROBE (Strengthening the Reporting of Observational Studies in Epidemiology) statement [22] and the CHERRIES (Checklist for Reporting Results of Internet E-Surveys) [23], and the qualitative and integration components drew on established thematic analysis [24] and mixed methods guidance [25], where applicable.

### Study Setting and Participants

The study was conducted at Shantou University Medical College from October to November 2025. Students used a self-developed AMTES to complete history-taking training for 3 assigned cases through text or voice interactions with virtual patients. After each session, they received automated scores, session summaries, and personalized feedback, including feedback on omitted or insufficiently explored aspects of the patient’s history and targeted suggestions for improvement. The AMTES platform was the same system evaluated in studies 1 and 2 [20,21], with the underlying large language model upgraded from DeepSeek-V2.5 to DeepSeek-V3 while the core training workflow and scoring logic remained unchanged.

**Figure 1:**
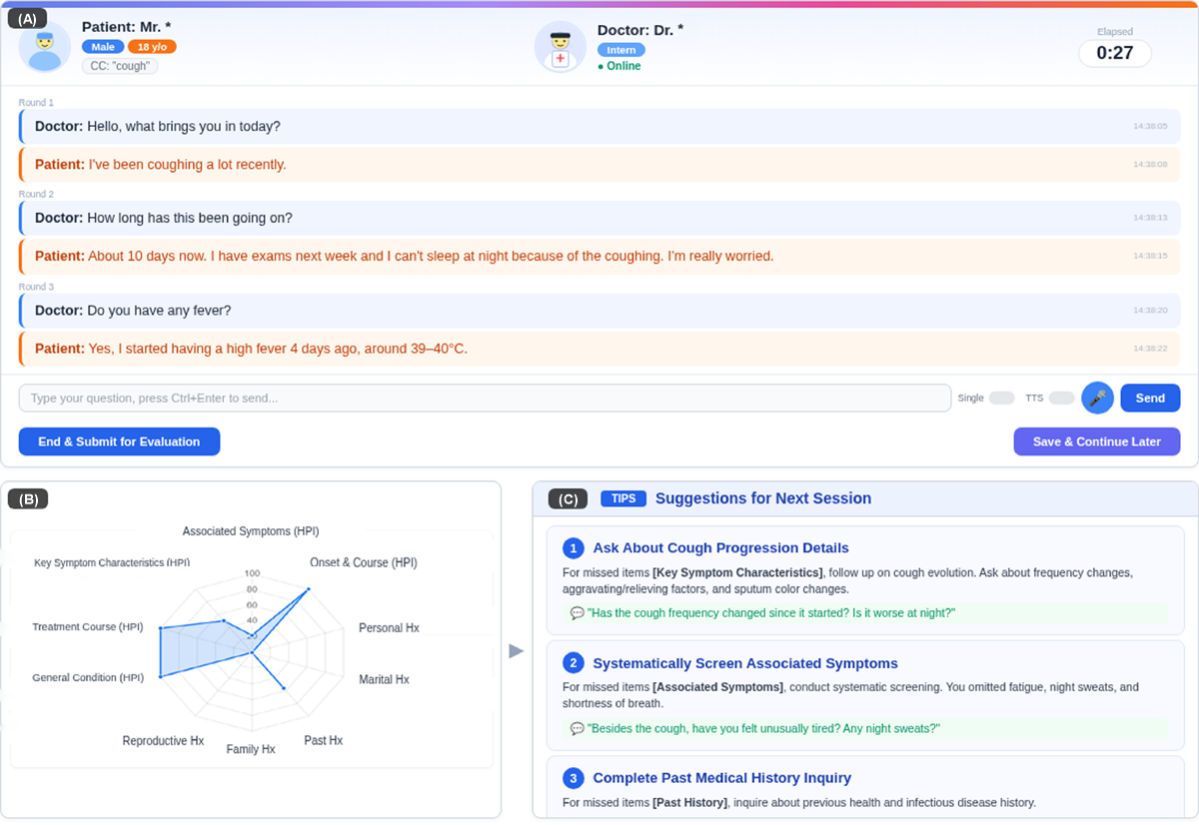
Example AI-powered medical history-taking training and evaluation system interface and feedback components available to students during and after a history-taking session. (A) The multiturn dialogue interface where students interact with a large language model–powered virtual patient through text or voice input. (B) A radar chart visualizing performance across multiple history-taking dimensions. (C) AI-generated personalized feedback identifying missed history items and providing specific suggested follow-up questions.

Participants were all undergraduate medical students enrolled in the Diagnostics course, who completed a postuse questionnaire after using AMTES for history-taking training as part of the course curriculum. Practice-log data showed 272 completed sessions from 66 students across 3 cases. Most students (62/66, 93.9%) completed all 3 required cases, and 30/66 (45.5%) undertook additional voluntary practice. Each session involved a mean of 108.0 (SD 37.6) dialogue turns and lasted 31.1 (SD 18.0) minutes, yielding a cumulative mean exposure of 128.4 minutes and 445.2 dialogue turns per student. The questionnaire was collected after the practice period ended, so participants responded after repeated hands-on exposure to the system; however, the anonymous questionnaire contained no identifiers for linking individual survey responses with practice-log variables. An example interface and feedback display of AMTES is shown in Figure 1.

### Recruitment, Sampling, and Eligibility Criteria

A whole-cohort sampling approach was used. Eligible students were all undergraduate medical students enrolled in the Diagnostics course during the study period who participated in the designated AMTES history-taking training. After the AMTES training and the summative history-taking examination had been completed and all course grades had been finalized and officially recorded, the study was introduced to the cohort during a subsequent regularly scheduled class session held several days later by a member of the research team who was also involved in course teaching. Students were informed of the study purpose and of the voluntary and anonymous nature of participation, and were told that the decision to participate or to decline would not affect their grades, course standing, or relationship with instructors. Because the questionnaire was anonymous and contained no identifiers, individual responses could not be linked to students; and because all grades had already been finalized and officially recorded, participation could not affect academic outcomes. Those who chose to participate completed the anonymous web-based questionnaire individually on their personal devices through a class messaging link during the same supervised class session; no reminders or incentives were used, and instructors did not monitor individual responses. Questionnaires with missing core outcome items or response patterns suggesting invalid completion (eg, identical responses across all conceptually distinct items) would have been excluded. None met these criteria. All 66 students had completed at least 1 assigned history-taking case, so every eligible student had substantive hands-on experience with AMTES on which to base their responses; the 4 students who did not complete all 3 assigned cases were retained because their completed sessions reflected meaningful engagement with the system. Thus, all 66 eligible students completed the questionnaire, yielding a 100% response rate.

### Questionnaire Design and Development

The questionnaire consisted of a baseline characteristics section; item sets on system experience, learning experience/intrinsic motivation, and overall acceptance; and 2 open-ended questions (C1 and C2), included to capture perceived strengths and areas for improvement not fully reflected in the structured item sets. C1 asked students to describe the greatest advantage of the system or what helped them most (“您认为这个系统最大的优点或给您带来最大帮助的是什么?”), and C2 asked them to identify the one aspect of the system that most needed improvement (“您认为这个系统最需要改进的一个方面是什么?”). Both prompts were deliberately valence-leading—C1 toward perceived benefits and C2 toward a single improvement priority—so the resulting theme frequencies are interpreted as respondent-nominated salience rather than prevalence or importance (see the Qualitative Analysis section). The baseline characteristics section captured participants’ age, sex, previous history-taking training experience, and previous experience with comparable AI systems. Age was entered numerically from the questionnaire categories, with the top category (“24 or above”) coded as 24, and was used together with sex for sample description and covariate-adjusted sensitivity analyses. Items on previous experience were retained for descriptive purposes only.

The quantitative item sets were each informed by established English-language scales and adapted to maintain contextual relevance and reduce redundancy. Candidate system-experience items (Block A; 12 items entering exploratory factor analysis [EFA], 5-point Likert scale) combined System Usability Scale (SUS)–informed usability items [26,27] with author-developed dialogue-quality items designed to capture system usability, interaction fluency, and dialogue experience. Learning experience/intrinsic motivation items (Block B; 11 items, 7-point Likert scale) were informed by selected Intrinsic Motivation Inventory subscales [28] covering interest/enjoyment, perceived competence, effort/importance, pressure/tension, perceived choice, and value/usefulness. Overall acceptance was assessed using 3 items rated on a 5-point Likert scale, covering intention to use, intention to recommend, and overall satisfaction.

To clarify the theoretical positioning, the acceptance model was treated as an exploratory, theory-informed framework rather than a formal test or validated adaptation of TAM or UTAUT, and the experience composites correspond only partially to established acceptance constructs. System experience (SUS-informed usability items together with author-developed dialogue-quality items) corresponds partially to perceived ease of use and effort expectancy as well as to system- and interaction-quality perceptions; learning experience/intrinsic motivation (Intrinsic Motivation Inventory subscales together with perceived value and confidence) corresponds partially to perceived usefulness and performance expectancy as well as to intrinsic motivation; and overall acceptance (intention to use, intention to recommend, and satisfaction) corresponds to behavioral intention and to Kirkpatrick Level 1 reaction and satisfaction. Because both composites are multifaceted and were constructed empirically through EFA, these correspondences denote partial conceptual alignment rather than validated construct equivalence (the mapping is summarized in Multimedia Appendix 1); accordingly, TAM and UTAUT were used as sensitizing frameworks to organize acceptance-related concepts, not as confirmatory latent-variable models to be statistically tested in this sample. The UTAUT constructs of social influence and facilitating conditions were not modeled: in a single, mandatory curricular deployment, these were expected to show limited between-student variability and fell outside the exploratory Level 1 scope. We therefore treat them as boundaries of this framework rather than as irrelevant factors, and note them as priorities for future, more fully specified acceptance models.

All items were translated into Chinese using a forward translation, cross-checking, and iterative revision process to improve semantic equivalence and expression consistency. The questionnaire was presented in bilingual format (Chinese as primary, English original as reference) to facilitate local readability while preserving item traceability during administration.

### Online Survey Administration

The questionnaire was administered as an anonymous closed web-based survey. Access was restricted to the eligible cohort and limited to 1 submission per respondent. The survey was distributed in person during a subsequent class session held several days after the summative history-taking examination had been completed and all grades had been finalized and officially recorded, ensuring that participation and responses could not affect academic outcomes. Although completion was voluntary, all core items were mandatory for those who chose to participate. Respondents could review and revise their answers before submission. Students accessed the survey individually on personal devices during a supervised on-site session, without course instructors observing responses in real time. The exported dataset was deidentified before analysis.

### Data Processing and Scoring

Reverse-worded items were reverse-scored before analysis. All 5-point and 7-point Likert items were then linearly rescaled to a 0-1 range to facilitate comparability across scales. Composite scores were calculated as the mean of the retained items within each set. Overall acceptance was calculated as the mean of intention to use, intention to recommend, and overall satisfaction.

### Statistical Analysis

#### Overview

All statistical analyses were performed using Python (version 3.12). All inferential tests were 2-sided, with the significance level set at α=.05. Upon data verification, the key variables used in this study had no missing values. Continuous variables were described using means and SDs, and categorical variables were reported as frequencies and percentages.

#### EFA

##### Factorability and Sampling Adequacy

Separate EFAs were conducted for Block A and Block B item sets to inform composite construction; the EFA was used for sample-specific data reduction to construct composites, not as formal scale validation. Factorability was assessed using Kaiser-Meyer-Olkin (KMO) measures of sampling adequacy [29] and Bartlett tests of sphericity [30]. Item evaluation considered item-level measure of sampling adequacy (MSA), factor loadings estimated using least-squares (minimum residual) factor extraction, and the consistency between item content and the intended construct. Items with MSA of <0.50 or factor loading of <0.40 were excluded from the final composite unless there was a strong conceptual rationale for retention. Factor retention for the final retained item sets was guided by parallel analysis [31,32]. Cronbach α was calculated for the final Block A and Block B item sets to assess internal consistency. Composite scores were calculated as the mean of the final retained items within each identified factor and used in subsequent regression analyses.

##### Supplementary Common-Method-Variance Check

Because quantitative data were from a single time point self-report questionnaire, Harman single-factor test was used as a supplementary common method bias check for common method variance [33]. Given that the acceptance items may reflect variance shared with the experience items, the Harman test excluding acceptance items was prioritized in interpretation.

##### Regression Models

To examine the association between experience-related measures and overall acceptance, we fitted a linear regression model with the overall acceptance composite as the dependent variable, and the retained composite scores derived from the EFA-informed item sets as independent variables. Standardized regression coefficients and *P* values based on heteroscedasticity-consistent SEs (type 3) [34] were reported, along with percentile bootstrap 95% CIs based on 5000 replications. Multicollinearity was assessed using variance inflation factors. Unique and shared variance decomposition was conducted as supplementary analyses.

Several sensitivity analyses were conducted to assess the robustness and directional consistency of the primary findings, including adjustment for age and sex, single-outcome models using use intention, recommendation intention, and overall satisfaction as separate dependent variables, and a fractional logit model for the bounded outcome [35]. A post hoc content-overlap check was also conducted by excluding the most overtly evaluative Block A items from the system-experience composite; Block A items were classified by the research team as acceptance-proximal (ease of use, confidence in use, feedback and report helpfulness, consultation fluency, and response usefulness) versus lower-overlap (functional integration, virtual-patient realism, nonmechanical interaction, and clinical-scenario realism) according to their semantic proximity to the acceptance items, and the item-level basis for this classification is reported in Multimedia Appendix 2.

#### Qualitative Analysis

Text responses to C1 and C2 were analyzed using codebook-oriented thematic analysis adapted for brief qualitative survey responses, drawing on Braun and Clarke’s [24] 6-phase approach as an organizing guide rather than as reflexive thematic analysis [36]. Because responses were brief written comments rather than interview transcripts, the analysis aimed to identify experiential patterns and optimization priorities rather than to develop deep process-oriented theory. Two AMTES research-team members jointly developed and refined the codebook and resolved coding disagreements through consensus; multicoding was permitted when a response described more than one distinct benefit or improvement direction; thus, although C2 asked respondents to nominate a single improvement priority, responses that raised more than one distinct actionable issue were multicoded for descriptive salience reporting rather than for prevalence estimation. Responses without an identifiable substantive theme were assigned a mutually exclusive “Vague/unclear” category. Translated quotes were verified by a bilingual researcher. Qualitative trustworthiness was supported by consensus-based codebook development, retention of verbatim representative quotes, transparent reporting of denominators and multicoding rules, and bilingual verification of translated quotes; because coding was consensus-based rather than independently blinded, interrater reliability statistics were not computed, and independent double-coding, member checking, and external auditing were not undertaken. Although reporting theme frequencies can imply a prevalence logic that Braun and Clarke [36] caution against for reflexive thematic analysis, transparent reporting of nomination frequencies can aid interpretation in codebook-oriented analysis of brief survey responses when the counts are framed as salience; accordingly, theme frequencies and all reported percentages should be interpreted as respondent-nominated salience rather than prevalence estimates or relative importance, especially because C1 and C2 were valence-leading prompts. We nonetheless acknowledge that frequency reporting in qualitative analysis should be interpreted cautiously and is intended here to enhance descriptive transparency for brief survey responses rather than to imply thematic importance.

#### Mixed Methods Integration

**Figure 2:**
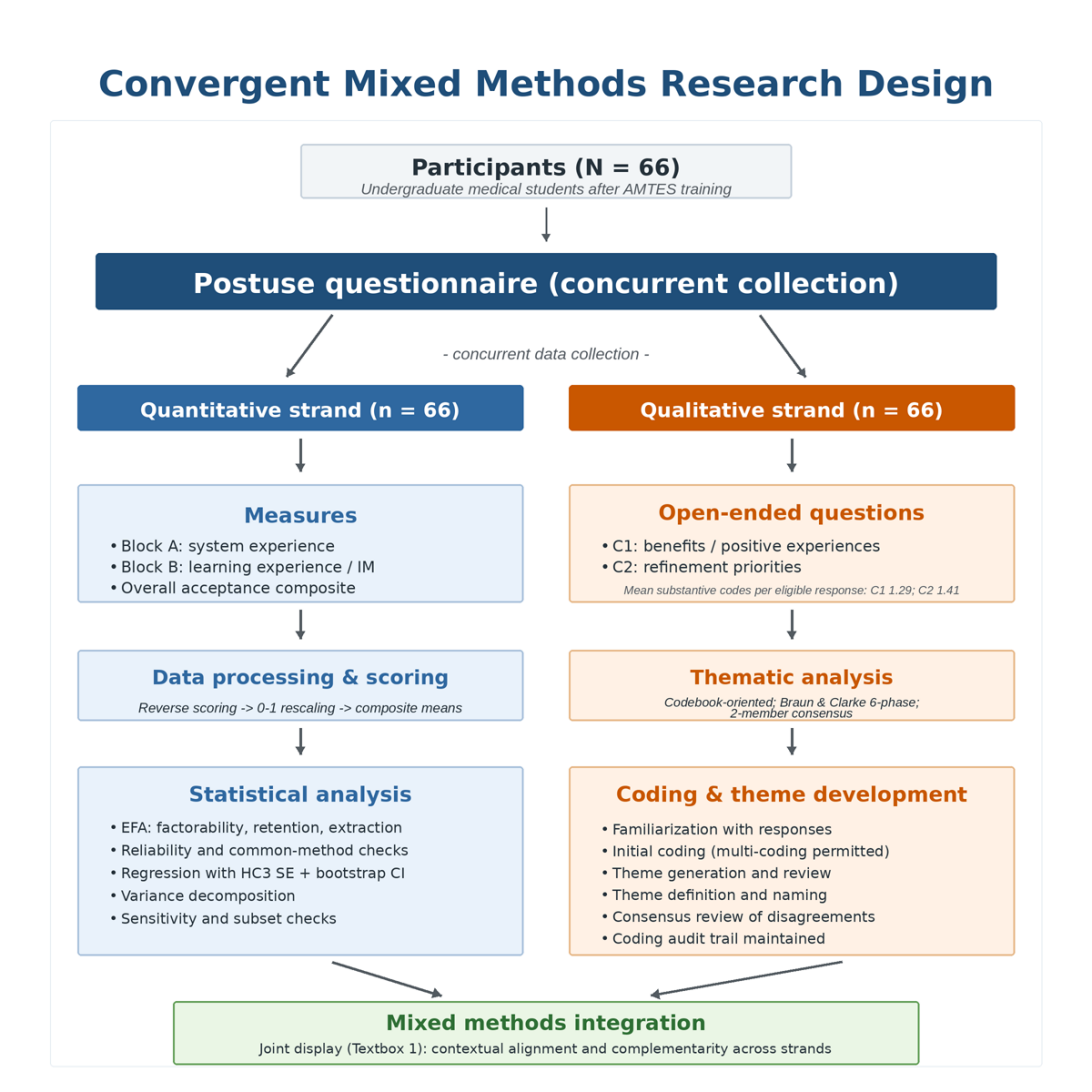
Convergent mixed methods design of the study. Quantitative and qualitative data were collected through the same postuse questionnaire, analyzed separately, and integrated at the results level through a joint display. AMTES: AI-powered medical history-taking training and evaluation system; EFA: exploratory factor analysis; HC3: heteroscedasticity-consistent standard errors (type 3); IM: intrinsic motivation.

This study used a convergent integration approach [25,37] in which quantitative and qualitative strands were analyzed separately and integrated at the results level through a joint display to identify contextual alignment, complementarity, and discrepancies; the overall design is summarized in Figure 2.

### Ethical Considerations

This study received ethical approval from the Ethics Committee of Shantou University Medical College (SUMC-2024-079) and followed the principles of the Declaration of Helsinki. AMTES training was part of the course, but participation in the anonymous postuse questionnaire and permission to use responses for research and publication were voluntary and covered by separate written informed consent obtained after summative examination and grade recording. All data were deidentified before analysis and stored on password-protected institutional servers accessible only to the research team; full free-text responses were not publicly released to protect participant privacy in this single-institution cohort. No financial compensation was provided.

## Results

### Participant Characteristics

**Figure 3:**
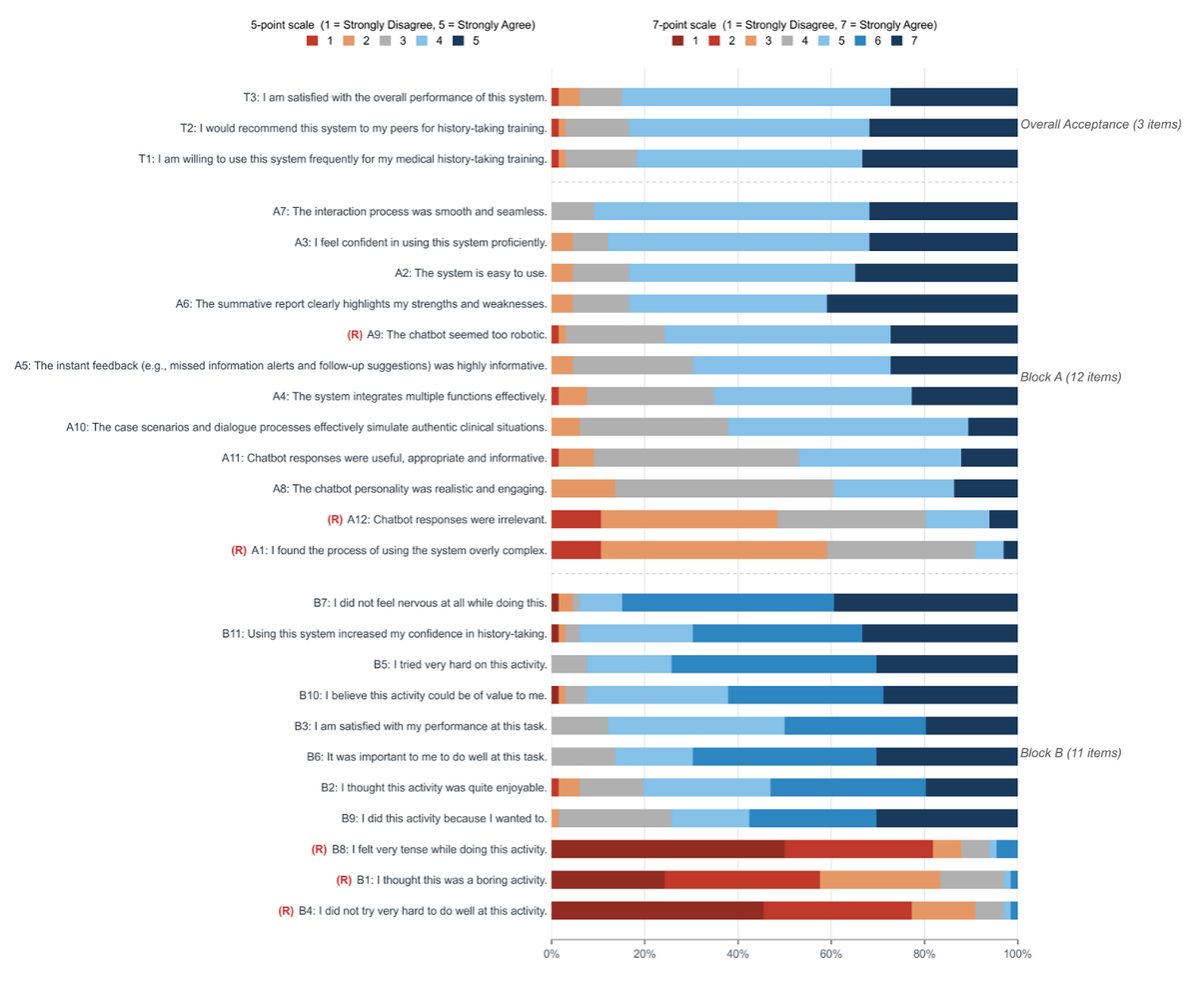
Item-level response distributions for overall acceptance, system experience, and learning experience/intrinsic motivation items among undergraduate medical students (N=66). Items are displayed as originally worded. Stacked bars show the percentage selecting each response category. Across most items, responses were concentrated in the favorable range, while reverse-worded items showed the opposite pattern.

All 66 undergraduate medical students enrolled in the Diagnostics course completed the questionnaire and were included in the analysis (response rate 100%). The mean age was 20.58 (SD 0.98) years; 33 (50%) were female, and 33 (50%) were male. Regarding previous experience, 22/66 (33.3%) students reported 1-2 previous history-taking practice occasions, 24/66 (36.4%) reported 3-4, and 20/66 (30.3%) reported 5 or more. For previous experience of using similar AI history-taking systems, 58/66 (87.9%) had never used one, 8/66 (12.1%) reported rare prior use, and none reported frequent prior use. Figure 3 presents item-level response distributions for overall acceptance, system experience, and learning experience/intrinsic motivation items. Responses were generally concentrated toward the favorable end of the scales, whereas reverse-coded items showed comparatively lower endorsement.

### EFA Results

#### Factorability and Factor Retention

**Figure 4:**
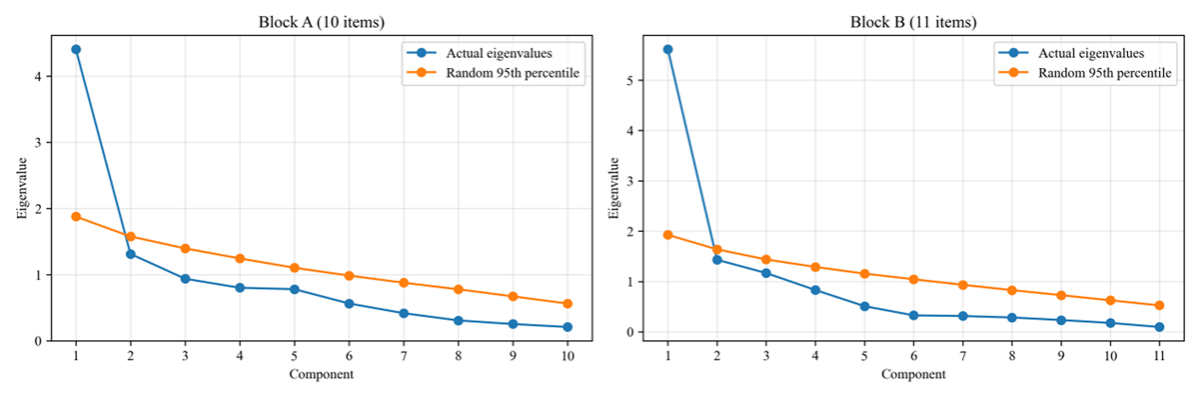
Parallel analysis results for factor retention in Block A and Block B (N=66). In both blocks, only the first observed eigenvalue exceeded the corresponding 95th percentile of the random eigenvalues, indicating a 1-factor solution.

Both Block A and Block B demonstrated adequate factorability. Block A had an overall KMO of 0.745, with a significant Bartlett test of sphericity (*χ*^2^_66_=317.66; *P*<.001); Block B had an overall KMO of 0.835, with a similarly significant Bartlett test (*χ*^2^_55_=460.97; *P*<.001). These results indicated that the interitem correlation structures in both sets were suitable for EFA. After item-level screening, parallel analysis of the final retained item sets further suggested retaining 1 factor for both Block A and Block B (Figure 4), indicating that both modules primarily reflected a single dominant dimension in this sample (Multimedia Appendix 3). Because the participant-to-item ratio was modest (approximately 5-6:1) and parallel analysis of the unscreened 12-item Block A set initially suggested a possible 2-factor structure, the 1-factor solutions are best regarded as provisional, exploratory composites for this sample rather than as evidence of a validated unidimensional scale.

#### Item Refinement and Internal Consistency

Block A initially included 12 candidate items. Two items were excluded during item screening because they did not meet the prespecified MSA and factor loading criteria, resulting in a final 10-item set. All 11 items in Block B were retained. The final item sets showed good internal consistency, with Cronbach α of 0.856 for Block A and 0.895 for Block B. The retained items were loaded onto a single factor within each block and were averaged to generate composite scores: A_total for system experience (Block A) and B_total for learning experience and intrinsic motivation (Block B), for use in subsequent regression analyses. Across the retained items, single-factor loadings ranged from 0.45 to 0.77 in Block A and from 0.42 to 0.84 in Block B, with corresponding communalities of approximately 0.20 to 0.60 and 0.17 to 0.70, respectively. Item-level MSA values, factor loadings, communalities, and retention decisions are reported in detail in Multimedia Appendix 3. Given the modest sample size, these composites should be interpreted as exploratory, and the factor structure may be subject to instability and overfitting; replication in larger samples is needed.

#### Supplementary Common-Method-Variance Check

Harman single-factor test showed that the first factor explained 38.3% of the variance after excluding acceptance items, and did not indicate a dominant single factor under this heuristic. Results were similar when all acceptance items were included, with the first factor explaining 41.7% of the variance. However, Harman single-factor test is an insensitive diagnostic, and residual common method variance cannot be excluded.

#### Regression Analyses

On the 0-1 rescaled composites, mean scores were 0.675 (SD 0.134) for A_total, 0.804 (SD 0.129) for B_total, and 0.771 (SD 0.194) for overall acceptance. The overall-acceptance composite showed high internal consistency (Cronbach α=0.938). Both the system-experience composite (A_total) and the learning-experience/intrinsic-motivation composite (B_total) were both significantly and positively associated with overall acceptance. A_total showed a stronger association (standardized β=.526, 95% CI 0.283-0.722; *P*<.001) than B_total (standardized β=.377, 95% CI 0.162-0.599; *P*=.002). The primary model explained 66.2% of the variance in overall acceptance (*R*^2^=0.662; adjusted *R*^2^=0.652; *F*_2, 63_=61.80; *P*<.001). Multicollinearity was low, with a variance inflation factor of 1.61 for both predictors. Additional variance-decomposition analyses are reported in Multimedia Appendix 4.

Sensitivity analyses yielded results consistent with the primary model. Adjustment for age and sex did not materially change the main associations, and similar patterns were observed across alternative outcome specifications (use intention, recommendation intention, and overall satisfaction) and a fractional logit model for the bounded acceptance outcome. In the post hoc content-overlap check, the lower-overlap system-experience composite remained positively associated with overall acceptance when modeled with B_total, although its independent association was attenuated in a partitioned model after the higher-overlap items were entered simultaneously, indicating that semantic content overlap contributed to the magnitude of the primary system-experience association (Multimedia Appendix 2).

### Qualitative Findings From Open-Ended Questions

#### Overview

Both open-ended questions yielded 66 responses. For C1 (perceived benefits), codebook-oriented thematic analysis with multicoding generated 2 main themes, 5 substantive subthemes, and 1 nonsubstantive category for vague responses. Multicoding was assigned to 18/66 (27.3%) C1 responses (18/65, 27.7% eligible substantive responses) and 18/66 (27.3%) C2 responses (18/59, 30.5% eligible substantive responses); theme percentages in Table 1 use the full response denominator (N=66), whereas the coding-density summaries reported in this paragraph use eligible substantive responses (excluding 1 vague C1 response and 7 nonspecific C2 responses). For C2 (refinement priorities), a multicoding approach was applied, generating 7 substantive themes and 1 nonsubstantive category; the 4 most-frequently-nominated themes (semantic understanding and intent recognition, contextual consistency across multiturn dialogue, conversational naturalness, and scoring accuracy and contextual scoring logic) were each cited by 21%-35% of all 66 respondents. Theme frequencies and representative quotes are summarized in Table 1.

**Table 1:** Summary of qualitative themes from open-ended questions (N=66)^a^.

Theme and subtheme				Responses, n (%)	Representative quote
**C1** **(Benefits)**					
	**Practice accessibility and** **feedback support**				
		Convenient and accessible self-directed practice	28 (42.4)		“It can be used anytime and anywhere, which is quite convenient” (P52)
		Low-pressure safe practice space	7 (10.6)		“I can think slowly without rushing to ask questions” (P27)
		Immediate feedback for self-assessment and gap identification	25 (37.9)		“I can review what was missed...clearly see where points were lost” (P43)
	**Competence development and clinical transition**				
		History-taking skill and process improvement	16 (24.2)		“Through repeated practice with this system, I became more familiar with the consultation process” (P58)
		Clinical realism and confidence building	8 (12.1)		“First get familiar with history-taking, then move on to SP conversations, and finally to clinical practice” (P26)
	**Nonsubstantive**				
		Vague or aesthetic-only response	1 (1.5)		—^b^
**C2** **(Refinement priorities)**					
	**Dialogue and interaction quality**				
		Semantic understanding and intent recognition	23 (34.8)		“Sometimes one needs to phrase the question very precisely for the system to understand” (P15)
		Contextual consistency across multiturn dialogue	17 (25.8)		“When asking about the situation three months earlier without restating the time frame, the patient answered about the current episode instead” (P51)
		Conversational naturalness	17 (25.8)		“I hope the AI patient can incorporate some normal conversational expressions and not appear so stiff” (P7)
	**Assessment logic**				
		Scoring accuracy and contextual scoring logic	14 (21.2)		“I had asked about certain points, but the system indicated I had not” (P35)
	**System and content resources**				
		System stability and performance	7 (10.6)		“Sometimes when many people are using it, you can’t log in” (P5)
		Feedback and report content	3 (4.5)		“The feedback could ideally provide a more complete case summary” (P55)
		Case library sufficiency	2 (3)		“The case set is limited; we tend to practice the same few cases repeatedly” (P42)
	**Nonsubstantive**				
		Blank, generic, or no specific suggestion	7 (10.6)		“None”/“Nothing for now”

^a^For both C1 and C2, multicoding was permitted: a single response could be assigned to more than one substantive subtheme. C1 subtheme percentages can sum to more than 100% across the full substantive subtheme set because 18/66 (27.3%) of responses were assigned more than one subtheme; C2 subtheme percentages similarly can sum to more than 100% across themes. The “Vague/unclear” nonsubstantive category is mutually exclusive with substantive subthemes. Participant identifiers (P1-P66) correspond to the survey sequence number. Percentages are based on total responses (N=66). All quotes were translated from Chinese. C2 percentages represent the frequency of student-identified suggestions, not dissatisfaction rates.

^b^Not available.

#### C1: Perceived Benefits and Positive Experiences

##### Theme 1: Practice Accessibility and Feedback Support

This theme, the most commonly identified in C1, captured students’ positive appraisal of AMTES as an accessible practice tool with helpful feedback.

###### Convenient and Accessible Self-Directed Practice

This was the most commonly identified benefit subtheme overall under multicoding (28/66, 42.4%), encompassing both anytime/anywhere convenience and the system’s role as an accessible practice channel for students with limited bedside opportunities. Students emphasized that AMTES extended practice opportunities beyond formal class time. For example, “It can be used anytime and anywhere, which is quite convenient; it reduces the tedious preparation before practice” (P52).

###### Immediate Feedback for Self-Assessment and Gap Identification

This was the second most commonly identified benefit subtheme (25/66, 37.9%). Students valued immediate feedback for identifying omissions and supporting reflection, with one student noting: “After the consultation, I can review what was missed, and there are records so I can clearly see the scoring and areas where points were lost” (P43).

###### Low-Pressure Safe Practice Space

Some students (7/66, 10.6%) described the system as a less stressful environment than SP or real-patient encounters, allowing them to think through questions more calmly (eg, P27 and P53). This subtheme also includes 2 responses where students described the system as allowing a slower, more deliberate thinking pace.

##### Theme 2: Competence Development and Clinical Transition

This theme captured students’ perceived value of AMTES for skill development and preparation for clinical encounters.

###### History-Taking Skill and Process Improvement

Some students (16/66, 24.2%) reported that repeated practice helped them become more familiar with the consultation structure and key questions before real history-taking. For example, “It helped me become more familiar with the consultation framework and key points before real history-taking” (P51).

###### Clinical Realism and Confidence Building

Students also linked AMTES to greater confidence in subsequent SP or clinical encounters, with some explicitly describing an “AI-to-SP-to-clinical” progression (8/66, 12.1%). For example, “You can first get familiar with history-taking, then move on to SP conversations, and finally to clinical practice” (P26).

One response (1/66, 1.5%) expressed a nonsubstantive aesthetic reaction without any identifiable benefit theme and was coded under the “Vague/unclear” nonsubstantive category.

#### C2: Student-Identified Refinement Priorities

##### Dialogue and Interaction Quality

###### Semantic Understanding and Intent Recognition

The most frequently cited refinement priority concerned the system’s ability to recognize question intent and respond appropriately (23/66, 34.8%). Students noted that semantically varied or context-dependent questions were not always well understood, suggesting room for improvement in context-sensitive intent disambiguation, for example, “sometimes one needs to phrase the question very precisely for the system to understand” (P15).

###### Contextual Consistency Across Multiturn Dialogue

Some students (17/66, 25.8%) suggested room for improvement in maintaining state across turns, noting occasional inconsistencies between earlier and later parts of the same conversation. For example, “when asking about the situation three months earlier without restating the time frame, the patient answered about the current episode instead” (P51).

###### Conversational Naturalness

Few students (17/66, 25.8%) suggested incorporating more natural conversational style and softening the AI patient’s response register, for example, “I hope the AI patient can incorporate some normal conversational expressions and not appear so stiff” (P7).

##### Assessment Logic

Scoring accuracy and contextual scoring logic (14/66, 21.2%): open-ended responses highlighted 2 opportunities to strengthen the alignment between student actions and system feedback: (1) more consistent recognition of history-taking content already covered by students, and (2) feedback that better reflects the clinical priorities of each case. As one student noted, “I had asked about certain points, but the system indicated I had not” (P35). These comments do not adjudicate scoring accuracy directly, but they are important because perceived mismatches may affect trust in automated feedback.

##### System and Content Resources

Three less frequent refinement priorities concerned system infrastructure and content: system stability and performance (7/66, 10.6%), mainly occasional access difficulty during peak use; feedback and report content (3/66, 4.5%), including requests for more comprehensive postsession summaries; and case library sufficiency (2/66, 3.0%), reflecting a desire for additional practice cases.

A total of 7 out of 66 (10.6%) responses provided no specific refinement suggestion; these included blank entries and brief generic remarks such as “None” or “Nothing for now.”

#### Mixed Methods Integration: Joint Display

Textbox 1 presents a joint display integrating the quantitative regression findings with the qualitative themes from C1 and C2, organized by the 2 core experience dimensions, to identify contextual alignment, complementarity, and cross-dimensional relationships not apparent from either strand alone.

**Joint display integrating quantitative and qualitative findings. Quantitative associations are described in plain language, with exact coefficients and 95% CIs reported in the text. C1 parent-theme percentages are respondent-level deduplicated: theme 1 was nominated by 49/66 (74.2%) students and theme 2 by 22/66 (33.3%) at the parent-theme level; because multicoding was permitted and 6 responses were coded to subthemes under both themes, these percentages are not mutually exclusive. Subtheme frequencies are reported in Table 1.**

**System experience (A_total)**

- Quantitative finding
  Stronger positive association with overall acceptance; robust across sensitivity analyses.
- Qualitative: benefits (C1)
  Practice accessibility and feedback support was the most frequently nominated benefit cluster, with convenient self-directed practice and immediate feedback as the 2 highest-frequency subthemes (Table 1).
- Qualitative: refinement priorities (C2)
  Four dialogue-quality and assessment dimensions emerged as leading refinement priorities: semantic understanding and intent recognition, contextual consistency across multi-turn dialogue, conversational naturalness, and scoring accuracy and contextual scoring logic (Table 1).
- Integration assessment
  Both strands show same-source alignment on system interaction as the more salient acceptance dimension. Qualitative data add 4 actionable refinement directions not derivable from the regression coefficient alone. Cross-strand analysis further suggests that perceived scoring-feedback discrepancies may be associated with reduced trust in the feedback function—a candidate explanatory pattern not apparent from the quantitative model alone.

**Learning experience/intrinsic motivation (B_total)**

- Quantitative finding
  Significant positive association with overall acceptance; weaker than system experience but directionally consistent across sensitivity analyses.
- Qualitative: benefits (C1)
  Competence development and clinical transition was the secondary benefit theme, with a subset of students spontaneously describing an AI-to-SP-to-clinical training progression (Table 1).
- Qualitative: refinement priorities (C2)
  Learning-motivation dimensions were infrequently nominated as refinement priorities in C2, suggesting that learning value was not a salient refinement concern in this cohort.
- Integration assessment
  Same-source alignment: the comparatively weaker quantitative association aligns with the lower qualitative salience of learning-related themes.
  Complementarity: learning-related concerns were infrequently nominated; interaction-level issues were more frequent, suggesting interaction quality may have been the more salient acceptance-related concern.

The joint display revealed contextual alignment between the 2 strands on system interaction as the more salient acceptance dimension, while qualitative data contributed complementary explanatory depth. Most notably, it suggested a hypothesized pathway whereby perceived scoring-feedback discrepancies may be associated with reduced feedback trust, a candidate cross-dimensional pattern not captured by the quantitative model. Because both strands were drawn from the same postuse questionnaire, their agreement reflects same-source alignment rather than independent triangulation; methodological implications of this are addressed in the Discussion.

## Discussion

### Principal Findings

As a Level 1–aligned evaluation of learner reactions following structured curricular use of AMTES, this study addressed 3 prespecified objectives. First, both system experience and learning experience/intrinsic motivation were significantly and positively associated with overall acceptance, with system experience showing the stronger of the 2 associations—a pattern that held across multiple sensitivity analyses. Second, qualitative analysis identified immediate feedback for self-assessment and convenient, accessible practice as the most frequently nominated benefits, while semantic understanding and intent recognition, contextual consistency across multiturn dialogue, and conversational naturalness emerged as the most frequently nominated refinement priorities. Third, integration of both strands through a joint display revealed contextual alignment between the quantitative and qualitative evidence and surfaced explanatory depth not apparent from the regression model alone. Most notably, this approach identified scoring-feedback alignment as a concrete optimization target and clarified how perceived assessment discrepancies may be associated with reduced trust in the feedback function. Unlike studies 1 and 2, which addressed technical reliability and educational effectiveness, respectively, this study addressed a distinct implementation question: which experience dimensions are associated with learner acceptance of AMTES in a structured curricular deployment, and where optimization efforts might be directed.

### System Experience and Learning Experience as Correlates of Overall Acceptance

Both system experience and learning experience/intrinsic motivation showed significant positive associations with overall acceptance. The descriptive variance decomposition in Multimedia Appendix 4 attributed unique explained variance to each predictor and substantial shared variance; given the predictor correlation, the modest sample size, and the exploratory composite construction, these attributions should be treated only as sample-specific context rather than durable evidence of predictor importance.

The proportion of variance explained by the primary model (*R*^2^=0.662) should be interpreted as an upper-bound, context-specific figure rather than as a precise effect size. Because the predictors and the outcome were measured by self-report within the same questionnaire, shared method variance is expected to inflate both the observed associations and the overall *R*^2^, and partial semantic overlap between some system-experience items and the acceptance composite likely contributes further. Consistent with this, content-overlap sensitivity analyses showed that a lower-overlap system-experience composite remained positively associated with acceptance, whereas in a partitioned model its independent association was attenuated once the higher-overlap items were entered (Multimedia Appendix 2). The coefficients should therefore be interpreted as context-specific associational estimates that may partly reflect measurement overlap, rather than as evidence of causal influence. At the same time, the persistence of a positive association in the lower-overlap sensitivity analyses suggests that the observed relationship is unlikely to be entirely an artifact of content overlap. The observation that system experience showed the stronger association holds within the original measurement specification rather than as evidence of a durable construct hierarchy. Because the design was cross-sectional, all reported relationships are associational, and the proposed mechanisms are candidate hypotheses for future longitudinal or experimental testing.

Substantively, this pattern is consistent with technology-acceptance research, in which perceived usefulness and effort-related interaction quality are central to acceptance [14,18,19]. In AI-supported medical and medical-education settings, emerging work further links trust and acceptance not only to perceived usefulness but also to technology-related factors affecting confidence in system outputs, interaction processes, and feedback quality [10,15]. One plausible interpretation is that when dialogue fluency, contextual continuity, or recognition accuracy require learners to work around the system, perceived educational value alone may not be sufficient to secure strong acceptance. The substantial shared variance between the 2 predictors (Multimedia Appendix 4) further indicates that the 2 experience dimensions overlap to a degree that necessitates joint interpretation rather than isolated analysis.

Qualitative data collected from the same postuse questionnaire provided contextual elaboration of the quantitative pattern. Among student-identified refinement priorities, 3 closely related dialogue-quality concerns emerged as primary student priorities: semantic understanding, contextual consistency across multiturn dialogue, and conversational naturalness. The coherence deficits students described (eg, needing to repeat subject and time qualifiers in follow-up questions) and semantic-understanding limitations (apparent keyword-matching reliance) map directly onto constructs measured by the system-experience items; when students must repeatedly work around recognition limitations, their system-experience ratings may be less favorable.

These findings operate at different levels: study 1 [20] assessed script-level technical accuracy by comparing system responses against case scripts and expert ratings, whereas this study captured user-perceived interaction quality—including multiturn contextual coherence and phrasing flexibility. The apparent inconsistency is therefore not a true contradiction. A response can match the case script yet still feel mechanical, poorly connected across turns, or misaligned with students’ scoring-feedback expectations.

A parallel pattern applies to scoring: study 1’s intraclass correlation coefficient reflected agreement with expert evaluators [20], whereas C2 captured students’ item-level perceptions of whether asked content was credited. C2 comments should therefore not be read as direct evidence that the system lacks expert-level scoring reliability; instead, they more likely reflect a combination of residual recognition mismatches, divergent student-expert interpretations of scoring criteria, and high learner expectations of near-perfect item-level agreement in this cohort. Prior medical and health care education literature indicates that learners judge assessment-generated feedback through perceived credibility, relevance, fairness, and alignment with educational purposes, and these judgments can influence whether feedback is used or discounted [38-40]. Because the questionnaire was administered anonymously, individual student comments about scoring or feedback accuracy could not be cross-checked against system logs at the item level. Even occasional discrepancies may disproportionately affect user trust in the feedback function [41]. Considered together, study 1 and this study provide complementary evidence: the former supports technical reliability, while the latter clarifies the mechanism by which user trust may still be challenged by perceived discrepancies in real educational use.

A further interpretive possibility is that interaction-quality concerns may spill over into perceptions of feedback credibility. When students perceive that the content they asked about was not credited, the core benefit of immediate feedback may appear less trustworthy, even if the underlying scoring framework is broadly reliable. Prior research suggests that medical students’ perceived trustworthiness of AI-generated feedback is not uniformly high, with ratings varying considerably across individuals depending on their prior attitudes toward AI [42]. This cross-theme relationship illustrates an explanatory contribution of qualitative analysis not captured by the 2 aggregated quantitative indicators. Specifically, it demonstrates how perceived assessment discrepancies may contribute to less favorable overall system-experience ratings.

### The Role of Learning Experience and Intrinsic Motivation

The learning-experience/intrinsic-motivation composite showed a statistically significant positive association with overall acceptance that was directionally consistent across sensitivity analyses. Qualitative findings were contextually aligned with this pattern: a substantial portion of benefit-related responses reflected students’ recognition of AMTES’s value in history-taking skill development and clinical confidence building, broadly consistent with prior reports that virtual patient systems can support clinical skill learning [8,9,43] and learner confidence [9].

A small subset of students spontaneously described an AI-to-SP-to-clinical progression, suggesting that AMTES was perceived as one component of a broader clinical competence-development sequence rather than as a stand-alone tool. This narrative is compatible with Kirkpatrick’s hierarchical logic, in which favorable Level 1 reactions may support later learning engagement and behavioral transfer, but these cross-sectional data do not test such downstream outcomes. Although based on this small subset of responses, the narrative suggests a candidate pedagogical implication: AMTES may be more clearly positioned within a structured pathway such as preclass preview, AI practice, SP assessment, and clinical clerkship.

Students’ refinement suggestions concentrated on interaction quality rather than learning value. Because C2 asked respondents to nominate a single improvement priority, this pattern indicates nomination salience rather than direct evidence that learning value was already adequate.

### Contextual Elaboration and Complementarity in Mixed Methods Integration

Because both strands were collected through the same postuse questionnaire, their agreement is best interpreted as same-source alignment plus qualitative elaboration rather than quasi-independent triangulation [25]. Integration nevertheless added explanatory depth beyond the quantitative model in 3 ways: qualitative data identified concrete refinement directions across 4 dialogue-quality and assessment dimensions; exposed cross-dimensional overlap whereby scoring-feedback concerns affected both perceived system function and learning value; and clarified how students situated AMTES within a broader AI-to-SP-to-clinical training sequence, a pedagogical expectation not apparent from Likert-scale data alone.

### Comparison With Prior Work

The finding that system-interaction quality and perceived learning value were both positively associated with acceptance is broadly consistent with research on AI-driven educational tools. Prior virtual patient studies show educational value [7-9,43,44], while newer large language model–based systems underscore the importance of dialogue authenticity, contextual coherence, and feedback quality [10-12].

The contribution of this study lies less in reiterating that AI-driven history-taking systems can be educationally useful and more in clarifying which interaction-level factors are associated with user acceptance during actual curricular use. Specifically, acceptance judgments in this context appeared to reflect not only perceived learning value but also students’ perceptions of whether the system recognized the intent of semantically varied follow-up questions, maintained coherence across turns, sustained a natural conversational style, and provided contextually appropriate scoring feedback. This adds implementation-level explanatory depth to a literature that has often emphasized overall effectiveness or satisfaction without identifying the interaction-level bottlenecks most relevant to routine adoption.

At the methodological level, the convergent mixed methods design with results-level integration through a joint display is consistent with mixed methods integration approaches in recent medical education guidance and broader methodological literature [25,37]. The joint display was particularly useful because it supported descriptive integration between the quantitative coefficient pattern and the qualitative emphasis on system-related benefits, while also helping identify refinement directions and cross-dimensional relationships not readily apparent from the quantitative analysis alone. This design may therefore provide a methodological example for future user evaluations of AI-driven educational technologies in clinical training.

### Practical Implications

The recommendations below are grounded in this single-institution undergraduate curricular deployment of AMTES; their transferability to other AI-driven history-taking systems, learner levels, or institutional settings requires independent evaluation. Based on the integrated evidence, this study proposes the following candidate priorities for refinement and pedagogical implementation within the current cohort.

First priority: enhance dialogue interaction quality. Students’ responses clustered around 3 closely related dialogue-quality dimensions: semantic understanding and intent recognition, contextual consistency across multiturn dialogue, and conversational naturalness. Addressing these issues may involve better intent recognition to reduce keyword dependence, stronger tracking of prior dialogue context, and more varied response phrasing to reduce the mechanical tone of simulated-patient responses. Because dialogue quality appears relevant to users’ immediate experience in natural-language virtual patient systems, these improvements are hypothesized to have relevance beyond AMTES itself, pending independent replication.

Second priority: bridge the gap between automated assessment and student expectations through contextual scoring logic. Technically, better recognition of consultation content despite wording variations may reduce mismatches between what students ask and what the system credits; scoring rules should also be adapted to case metadata so that age-, sex-, and context-dependent items are evaluated only when clinically appropriate. Pedagogically, guided postsession debriefing, such as instructor-led review of AI-generated feedback, may help students understand the scoring logic and turn perceived discrepancies into learning opportunities.

Third priority: strengthen learning value through curricular integration and content expansion. AMTES may be particularly useful when embedded within a structured training sequence rather than used as a stand-alone activity. Positioning it within a pathway such as preclass preview, AI practice, SP assessment, and clinical clerkship may clarify its pedagogical role and strengthen perceived learning value, although this recommendation draws on a relatively small subset of student responses. Case-library expansion may also be considered as a secondary improvement to support repeated practice, although this was a low-salience request in this cohort.

### Limitations

This study has several limitations. First, the sample was drawn from a single institution and a single course cohort, which limits the generalizability of the findings to other institutions, learner levels, and educational contexts. The modest sample size means that factor-analytic results should be treated as exploratory and sample-specific rather than as formal psychometric validation; replication in larger and more diverse samples is needed before the observed patterns can be interpreted with confidence. Given the developer/evaluator dual role disclosed in the Conflicts of Interest section, findings should be interpreted with this context in mind. Several authors were involved in developing, deploying, teaching with, and evaluating AMTES. To mitigate the associated bias, the questionnaire was administered anonymously and only after the summative examination and after all grades had been finalized and officially recorded, with no linkage to grades and with no instructor access to identifiable responses; multiple sensitivity analyses, including post hoc analyses clearly labeled as such, were reported transparently; and student-identified critical feedback was deliberately retained and reported transparently irrespective of valence. Nonetheless, the primary outcomes were self-reported, the qualitative coding was performed by research team members who were not blinded to the system’s authorship and without an independent external auditor, and no independent third-party evaluation of AMTES was undertaken; independent external evaluation is an important direction for future work.

Second, quantitative data were collected via a self-report questionnaire at a single time point, introducing the possibility of social desirability bias and common method bias. Although procedural safeguards, including postexamination administration, explicit separation from course grading, and anonymized data handling, were implemented, the Harman single-factor test is an insensitive diagnostic that cannot rule out common method variance. Therefore, the observed associations and explained variance may be partly inflated by shared method variance, and future studies should incorporate procedural and statistical remedies such as temporal or source separation of predictors and outcomes or marker-variable techniques. Some Block A items also shared evaluative content with the acceptance composite, so the observed association between system experience and overall acceptance may partly reflect content overlap. Finally, acceptance reflected self-reported intention and satisfaction rather than observed adoption behavior, and the anonymous questionnaire format precluded person-level linkage between acceptance judgments and actual usage data. Several considerations support the plausibility of the 100% response rate and mitigate concern about coercion: the cohort was small and sampled as a whole; every student had already completed at least one history-taking case and therefore had direct experience of AMTES on which to base their responses; the survey was brief and administered in person during a single supervised session; and it was conducted only after the summative examination had ended and all grades had been finalized and officially recorded, several days later, so that participation could not affect academic outcomes. Participation was voluntary and anonymous, with no linkage to grades, and students were told that declining carried no academic consequence. We nonetheless acknowledge that the study was introduced by a member of the research team who was also involved in course teaching, and that a supervised classroom setting may carry some perceived expectation to participate; although the anonymous administration (which prevented any instructor from linking responses to individual students) and the postgrading timing were designed to minimize this, a residual perception of obligation cannot be entirely excluded.

Third, the qualitative component was constrained by the brevity of open-ended responses, which limited thematic depth and precluded saturation; future research incorporating semistructured interviews or focus groups could yield richer experiential narratives. Student-reported scoring discrepancies were not independently adjudicated against expert review at the item level, meaning the qualitative data could not distinguish actual system errors from misunderstanding of scoring criteria or expectation-based perceptions. As a cross-sectional study, this work cannot address longitudinal changes in acceptance or sustained real-world adoption; future linkage-enabled studies are needed to examine whether postuse acceptance predicts subsequent voluntary engagement and whether iterative system refinement translates into improved user experience over time.

### Conclusions

To our knowledge, relatively few prior studies have used a convergent mixed methods design to examine both the experience dimensions associated with user acceptance and the underlying experiential patterns in AI-powered history-taking training systems. This study contributes to this small but growing literature. Unlike prior work that has primarily addressed technical reliability or educational effectiveness, this study extends the focus to implementation-level acceptance. The findings suggest that, in this cohort and within the study’s measurement specification, system interaction quality showed a stronger association with acceptance than with perceived learning value during structured curricular use of AMTES. This distinction carries practical implications for system developers, who may need to prioritize interaction-level refinements over feature expansion, and for educators, who may benefit from explicitly positioning such systems within structured training pathways.

From a broader perspective, these findings contribute to the growing evidence base on AI-driven educational technologies by suggesting a plausible mechanism linking natural-language interaction quality to user acceptance. Specifically, perceived coherence deficits and scoring-feedback discrepancies may be associated with reduced trust in the feedback function and, by extension, with less favorable overall acceptance even when educational value is recognized. The convergent mixed methods design, with results-level integration through a joint display, was particularly useful in surfacing this cross-dimensional relationship, which would have remained invisible to purely quantitative approaches. For the field more broadly, dialogue coherence, semantic flexibility, and scoring-feedback alignment may warrant attention as candidate optimization priorities in natural-language virtual patient systems, pending replication in diverse settings. Larger multicenter and longitudinal studies are needed to test the generalizability of these patterns and to examine whether iterative improvements in interaction quality translate into measurable and sustained gains in learner acceptance and downstream training outcomes.

## Data Availability

Deidentified quantitative survey data, case-level practice-log summary data, and the Python analysis scripts used for data cleaning and statistical analysis are available from the corresponding author on reasonable request. Because raw free-text responses from this single-institution cohort may contain contextual details that increase the risk of reidentification, those materials are not publicly released; a redacted version may be made available on reasonable request, subject to institutional and ethics requirements.

## Appendix

Multimedia Appendix 1 Construct mapping between study composites and established acceptance frameworks.

Multimedia Appendix 2 Sensitivity analyses.

Multimedia Appendix 3 Item-level sampling adequacy, factor loadings, communalities, and item retention.

Multimedia Appendix 4 Supplementary variance-decomposition analyses.

## Abbreviations

AI-VP: AI-powered virtual patient
AMTES: AI-powered medical history-taking training and evaluation system
CHERRIES: Checklist for Reporting Results of Internet E-Surveys
EFA: exploratory factor analysis
KMO: Kaiser-Meyer-Olkin
MSA: measure of sampling adequacy
SP: standardized patient
STROBE: Strengthening the Reporting of Observational Studies in Epidemiology
SUS: System Usability Scale
TAM: Technology Acceptance Model
UTAUT: Unified Theory of Acceptance and Use of Technology
VP: virtual patient
