# Supplementary material for: User Acceptance of an AI-Powered Medical History–Taking Training System Among Undergraduate Medical Students: Mixed Methods Study
Source: JMIR Med Educ. 2026 Jul 21;12:e101425. doi: 10.2196/101425 (PMC13439043; doi:10.2196/101425)
Supplement: Supplementary file 1 [file mededu_v12i1e101425_app1.pdf]

Multimedia Appendix 1: Construct Mapping Between Study Composites and Established Acceptance Frameworks

This appendix summarizes how the empirically constructed study composites correspond to constructs from established acceptance and training-evaluation frameworks. The acceptance model was treated as an exploratory, theory-informed framework: TAM and UTAUT were used as sensitizing frameworks to organize acceptance-related concepts, not as confirmatory latent-variable models to be statistically tested in this sample. The correspondences below denote partial conceptual alignment, not validated construct equivalence.

**Observed composite (this study)**	**Included item content**	**Partial conceptual correspondence to prior frameworks**
System experience (A_total) — an EFA-informed system-interaction composite	SUS-informed usability items (ease of use, confidence in use, functional integration) and author-developed dialogue-quality items (interaction fluency, instant-feedback and report helpfulness, virtual-patient realism/engagement, clinical-scenario realism, non-mechanical interaction, response usefulness)	TAM perceived ease of use; UTAUT effort expectancy; system-quality / interaction-quality and information/feedback-quality perceptions
Learning experience / intrinsic motivation (B_total) — an EFA-informed learning-value/motivation composite	Intrinsic Motivation Inventory subscales (interest/enjoyment, perceived competence, effort/importance, pressure/tension, perceived choice, value/usefulness) and a perceived-confidence item	TAM perceived usefulness; UTAUT performance expectancy; intrinsic motivation (self-determination perspective)
Overall acceptance	Intention to use, intention to recommend, and overall satisfaction	TAM/UTAUT behavioral intention; Kirkpatrick Level 1 reaction and satisfaction
Not modeled (framework boundaries)	Social influence and facilitating conditions were not measured	UTAUT social influence and facilitating conditions; not modeled because a single, mandatory curricular deployment was expected to show limited between-student variability and these fell outside the exploratory Level 1 scope; flagged as priorities for future, more fully specified acceptance models

Note. Composites were derived by exploratory factor analysis for sample-specific data reduction, not as validated scales. Item-level loadings, communalities, and retention decisions are reported in Multimedia Appendix 3 .
