# Supplementary material for: User Acceptance of an AI-Powered Medical History–Taking Training System Among Undergraduate Medical Students: Mixed Methods Study
Source: JMIR Med Educ. 2026 Jul 21;12:e101425. doi: 10.2196/101425 (PMC13439043; doi:10.2196/101425)
Supplement: Supplementary file 2 [file mededu_v12i1e101425_app2.pdf]

Multimedia Appendix 2: Sensitivity Analyses

Table 1. Regression model with demographic covariates.

Predictor	Standardized β (bootstrap 95% CI)	*P* value[a]
A_total	0.559 (0.338–0.751)	<.001
B_total	0.363 (0.15–0.575)	0.004
Age	0.13 (-0.078–0.349)	0.28
Sex (Male vs Female)	0.025 (-0.114–0.187)	0.786
Model fit: R^2 = 0.68; adjusted R^2 = 0.66; $F(4,61)$ = 32.48; P <.001		

[a]*P* values were based on robust standard errors.

This model examined whether the associations of A_total and B_total with overall acceptance remained robust after adjusting for age and sex. Both experience-related predictors remained positively associated with overall acceptance, whereas age and sex were not statistically significant covariates.

Table 2. Single-outcome sensitivity analyses.

Outcome	A_total, standardized β (95% CI)	*P* value	B_total, standardized β (95% CI)	*P* value	Adjusted R^2
Use intention	0.469 (0.172– 0.7)	.004	0.34 (0.093–0.587)	.01	0.516
Recommendation intention	0.477 (0.28–0.659)	<.001	0.432 (0.224–0.625)	<.001	0.657
Overall satisfaction	0.541 (0.275–0.747)	<.001	0.298 (0.074–0.533)	.018	0.566

These models examined whether the primary findings were consistent when the three components of the overall-acceptance composite were analyzed separately. A_total and B_total remained positively associated with each individual acceptance outcome.

Table 3. Fractional logit sensitivity analysis for bounded overall acceptance.

Predictor	Base model coefficient (95% CI)	*P* value	Adjusted model coefficient (95% CI)	*P* value
A_total	5.365 (3.367–7.362)	<.001	5.623 (3.818–7.429)	<.001
B_total	3.695 (1.991–5.398)	<.001	3.559 (1.877–5.240)	<.001
Age	—	—	0.137 (-0.081–0.355)	.217
Sex (Male vs Female)	—	—	0.113 (-0.261–0.486)	.554

This analysis examined whether the primary findings were robust to using a model appropriate for a bounded 0 – 1 outcome. A_total and B_total remained positively associated with overall acceptance in both the base and demographic-adjusted models.

Table 4. Sensitivity to evaluative-content overlap in Block A.

Model[a]	Model specification	Focal predictor	Standardized β (bootstrap 95% CI)	P value	R^2
Original model	A_total + B_total	A_total	0.526 (0.283– 0.722)	<.001	0.662
Lower-overlap model	A_no_overlap + B_total	A_no_overlap	0.333 (0.095–0.539)	.021	0.574
Overlap-only model	A_overlap_only + B_total	A_overlap_only	0.584 (0.364– 0.771)	<.001	0.693
Partitioned model	A_no_overlap + A_overlap_only + B_total	A_no_overlap	-0.001 (-0.265– 0.237)	.994	0.693
Lower-overlap adjusted model	A_no_overlap + B_total + age + sex	A_no_overlap	0.379 (0.165–0.582)	.007	0.593

[a]A_total is the original 10-item Block A system-experience composite. A_no_overlap included A4, A8, A9, and A10, representing lower-overlap items related to functional integration, virtual-patient realism/engagement, non-mechanical interaction, and clinical-scenario realism. A_overlap_only included A2, A3, A5, A6, A7, and A11, representing items more semantically proximal to acceptance-related judgments, including ease of use, confidence in use, feedback helpfulness, report helpfulness, consultation fluency, and response usefulness/appropriateness/informativeness. All models included B_total.

This post hoc analysis examined whether the association between system experience and overall acceptance was driven by Block A items with wording semantically close to acceptance. A_no_overlap retained the 4 lower-overlap Block A items, whereas A_overlap_only retained the 6 items judged to be more semantically proximal to use intention, recommendation, or satisfaction.

After excluding the 6 higher-overlap Block A items, the lower-overlap composite remained positively associated with overall acceptance, suggesting that the primary system-experience association was not fully explained by evaluative-content overlap. However, in the partitioned model, A_no_overlap was no longer independently associated with overall acceptance when A_overlap_only was entered simultaneously, indicating that content overlap may still partly contribute to the observed association.
