# Supplementary material for: User Acceptance of an AI-Powered Medical History–Taking Training System Among Undergraduate Medical Students: Mixed Methods Study
Source: JMIR Med Educ. 2026 Jul 21;12:e101425. doi: 10.2196/101425 (PMC13439043; doi:10.2196/101425)
Supplement: Supplementary file 3 [file mededu_v12i1e101425_app3.pdf]

Multimedia Appendix 3: Item-Level Sampling Adequacy, Factor Loadings, Communalities, and Item Retention

Table 1. Item-level MSA, single-factor loadings, communalities, and retention decisions for candidate Block A and Block B items.

Item	MSA	Absolute factor loading	Communality (h^2)	Retained
Block A: System Experience (12 EFA items → 10 retained)				
A1: I found the process of using the system overly complex.	0.498	0.140	0.020	No
A2: The system is easy to use.	0.734	0.593	0.352	Yes
A3: I feel confident in using this system proficiently.	0.793	0.663	0.440	Yes
A4: The system integrates multiple functions effectively.	0.890	0.774	0.599	Yes
A5: The instant feedback (e.g., missed information alerts and follow-up suggestions) was highly informative.	0.699	0.615	0.378	Yes
A6: The summative report clearly highlights my strengths and weaknesses.	0.735	0.668	0.446	Yes
A7: The interaction process was smooth and seamless.	0.770	0.464	0.215	Yes
A8: The chatbot personality was realistic and engaging.	0.759	0.660	0.436	Yes
A9: The chatbot seemed too robotic.	0.768	0.449	0.202	Yes
A10: The case scenarios and dialogue processes	0.770	0.549	0.301	Yes

effectively simulate authentic clinical situations.				
A11: Chatbot responses were useful, appropriate and informative.	0.702	0.662	0.438	Yes
A12: Chatbot responses were irrelevant.	0.662	0.357	0.127	No
Block B: Learning Experience / Intrinsic Motivation (11 items → 11 retained)				
B1: I thought this was a boring activity.	0.878	0.628	0.394	Yes
B2: I thought this activity was quite enjoyable.	0.884	0.702	0.493	Yes
B3: I am satisfied with my performance at this task.	0.941	0.839	0.704	Yes
B4: I did not try very hard to do well at this activity.	0.749	0.537	0.288	Yes
B5: I tried very hard on this activity.	0.839	0.751	0.564	Yes
B6: It was important to me to do well at this task.	0.910	0.730	0.533	Yes
B7: I did not feel nervous at all while doing this.	0.706	0.491	0.241	Yes
B8: I felt very tense while doing this activity.	0.634	0.416	0.173	Yes
B9: I did this activity because I wanted to.	0.920	0.776	0.602	Yes
B10: I believe this activity could be of value to me.	0.801	0.782	0.612	Yes
B11: Using this system increased my confidence in history-taking.	0.798	0.727	0.529	Yes

Note. MSA = measure of sampling adequacy. Loadings are absolute single-factor loadings estimated by least-squares (MINRES) factor extraction. For a one common-factor solution, the communality (h^2) equals

the squared single-factor loading and represents the proportion of item variance accounted for by the retained factor. During item-level screening, A1 was excluded for MSA <0.50 (MSA=0.498) and A12 for factor loading <0.40 (loading=0.357). Among the retained items, communalities ranged from approximately 0.20 to 0.60 in Block A and from 0.17 to 0.70 in Block B. Because the sample was modest (N=66) and the participant-to-item ratio was approximately 5–6:1, these item-level estimates should be interpreted as exploratory and sample-specific rather than as formal scale validation.

Table 2. Parallel analysis eigenvalue comparison for the retained item sets.

Component	Actual eigenvalue	Random-data 95th percentile	Retained
Block A (10 items, post item-level screening)			
1	4.409	1.878	Yes
2	1.314	1.577	No
3	0.939	1.398	No
4	0.802	1.245	No
5	0.781	1.105	No
Block B (11 items)			
1	5.612	1.917	Yes
2	1.432	1.635	No
3	1.169	1.439	No
4	0.832	1.287	No
5	0.511	1.157	No

In the initial 12-item Block A set, parallel analysis suggested a possible two-factor structure. Item-level review identified one item with MSA <0.50 and one item with factor loading <0.40. After excluding these items, parallel analysis of the final 10-item set supported a one-factor solution. Because the purpose of the exploratory factor analysis was exploratory composite construction rather than formal scale validation, the final 10-item solution was used for A_total. Parallel analysis was performed with 1000 random datasets; a factor was retained when its actual eigenvalue exceeded the 95th-percentile eigenvalue from the random-data reference distribution. In both blocks, only the first observed eigenvalue exceeded the corresponding 95th percentile of the random eigenvalues, indicating a one-factor solution.
