# Supplementary material for: User Acceptance of an AI-Powered Medical History–Taking Training System Among Undergraduate Medical Students: Mixed Methods Study
Source: JMIR Med Educ. 2026 Jul 21;12:e101425. doi: 10.2196/101425 (PMC13439043; doi:10.2196/101425)
Supplement: Supplementary file 4 [file mededu_v12i1e101425_app4.pdf]

Multimedia Appendix 4: Supplementary Variance-Decomposition Analyses

To provide descriptive context for the primary regression model, we examined the p between A_total (system experience) and B_total (learning experience/intrinsic motivation) in explaining overall acceptance. These analyses were conducted as supplementary descriptive analyses and should not be interpreted as stable evidence that one predictor is generally more important than the other.

A_total and B_total were moderately correlated (r=.615, P<.001). Both predictors also showed strong zero-order correlations with overall acceptance (A_total: r=.758, P<.001; B_total: r=.700, P<.001). In the full model, A_total and B_total together explained 66.2% of the variance in overall acceptance. Of this explained variance, 26.0% was unique toA_total, 13.4% was unique to B_total, and 60.6% was shared by the 2 predictors.
